# Age-adjusted high-dose chemotherapy and autologous stem cell transplant in elderly and fit primary CNS lymphoma patients

**DOI:** 10.1186/s12885-019-5473-z

**Published:** 2019-03-29

**Authors:** Elisabeth Schorb, Juergen Finke, Gabriele Ihorst, Benjamin Kasenda, Heidi Fricker, Gerald Illerhaus

**Affiliations:** 1 Department of Hematology, Oncology and Stem Cell Transplantation, Faculty of Medicine, University of Freiburg, Hugstetter Straße 55, 79106 Freiburg, Germany; 2 Clinical Trials Unit, Faculty of Medicine and Medical Center, University of Freiburg, Freiburg, Germany; 3 Clinic of Hematology, Oncology and Palliative Care, Klinikum Stuttgart, Stuttgart, Germany; 4 Department of Medical Oncology & Institute of Clinical Epidemiology and Biostatistics, University Hospital Basel, Basel, Switzerland

**Keywords:** Primary central nervous system lymphoma (PCNSL), High-dose chemotherapy (HDT), Autologous stem cell transplantation (ASCT), Elderly patients

## Abstract

**Background:**

Primary central nervous system lymphoma (PCNSL) is a diffuse large B-cell lymphoma (DLBCL) confined to the central nervous system (CNS) with rising incidence among patients > 65 years. Although elderly patients are able to tolerate aggressive systemic chemotherapy, previous studies have demonstrated inferior outcomes for patients who present with a poor performance status (PS) and older age. Usually, intensive treatment approaches including high-dose chemotherapy followed by autologous stem cell transplantation (HDT-ASCT) are only offered to patients younger than 65–70 years of age.

**Methods:**

This is an open-label, multicentric, non-randomized, single arm phase II trial. We will recruit 51 immuno-competent patients with newly diagnosed PCNSL from 12 German centers. The objective is to investigate the efficacy of age-adapted induction treatment followed by HDT-ASCT. All enrolled patients will undergo induction chemotherapy consisting of 2 cycles of rituximab 375 mg/m^2^/d (days 0 & 4), methotrexate 3.5 g/m^2^ (d1), and cytarabine 2 × 2 g/m^2^/d (d2–3) every 21 days. After 2 cycles of induction chemotherapy, patients achieving at least stable disease will undergo HDT-ASCT with busulfan 3.2 mg/kg/d (days − 7-(− 6)) and thiotepa 5 mg/kg/d (days − 5-(− 4)) followed by autologous stem cell transplantation. The primary endpoint of this study is 1-year progression-free survival (PFS). Secondary endpoints include PFS, overall survival, treatment response and treatment-related morbidities. Minimal follow-up after treatment completion is 12 months.

**Discussion:**

Current treatment options for PCNSL have improved over the last years, resulting in the potential to achieve durable remission or cure in patients < 70 years. Age alone may not be the only criterion to select patients for this effective treatment approach and probably many elderly patients are undertreated just because of advanced age. There have been no multicentre trials investigating this curative treatment concept in elderly and fit PCNSL patients so far. We aim to answer whether HDT-ASCT is feasible and effective in fit patients > 65 years with newly-diagnosed PCNSL.

**Trial registration:**

German clinical trials registry DRKS00011932 registered 18 August 2017.

**Electronic supplementary material:**

The online version of this article (10.1186/s12885-019-5473-z) contains supplementary material, which is available to authorized users.

## Background

Primary central nervous system lymphoma (PCNSL) is defined as a diffuse large B-cell lymphoma (DLBCL) confined to the central nervous system (CNS). [1] Elderly patients over 65 years are more commonly affected with a rising incidence in this age group [2, 3]. Standard treatment consists of high-dose methotrexate (HD-MTX) based chemotherapy to induce lymphoma remission followed by consolidation therapy. The IELSG20 trial established the combination of HD-MTX and high-dose cytarabine (HD-AraC) by demonstrating increased response as well as prolonged failure free survival and overall survival (OS) at 3 years [4]. Regarding consolidation therapy, HDT-ASCT with carmustine (BCNU) and thiotepa as well as with busulfan and thiotepa has been shown to be feasible and highly effective in different clinical trials [5–9]. The randomized IELSG32 trial of the International Extranodal Lymphoma Study Group (IELSG) was designed to determine the best induction treatment (1st randomization) and the best consolidation treatment (2nd randomization) and compared three different induction chemo(immuno)therapy regimens in patients with newly diagnosed PCNSL. This trial clearly showed that the MATRix combination (MTX, AraC, thiotepa (TT), and rituximab) followed by consolidation therapy significantly improved outcomes in eligible patients aged 70 years or younger [10, 11] and is now regarded as a new treatment standard in many countries. However, the IELSG32 trial was restricted to patients aged 70 years or younger with Eastern Co-operative Oncology Group Performance Status (ECOG PS) ≤2 (and < 65 years with ECOG PS ≤3), whereas many patients encountered in routine practice are older than those treated in the IELSG32 trial. Given that previous studies [9, 12, 13] have demonstrated inferior outcomes for lymphoma patients who present with a poor PS and older age, clinicians may be concerned about using such an intensive regimen in this patient cohort with special challenges like multiple comorbidities and polypharmacy. However, age alone may not be the only criterion to select patients and probably many elderly patients are undertreated just because of advanced age. Thus, an age-adapted treatment regimen is urgently needed to offer a curative treatment approach to this special treatment subgroup of patients.

A recently published retrospective analysis of elderly PCNSL patients undergoing HDT-ASCT showed promising results both in first-line and second or subsequent line of treatment [14]. The MARiTA trial, a pilot study investigating feasibility and efficacy of 2 cycles of induction treatment with rituximab, HD-MTX and AraC followed by HDT-ASCT in PCNSL patients > 65 years of age has recently completed recruitment (DRKS-ID 00008900). Prospective trials are needed to better define eligibility for this approach and to further improve therapeutic options in this unique subgroup of patients. In the herein proposed multicenter phase II study we want to investigate efficacy, feasibility, and safety of age-adapted induction treatment followed by HDT-ASCT in elderly PCNSL patients (> 65 years).

## Methods

### Study design

This is an open-label, multicentric, non-randomized, single arm phase II trial using a Fleming one stage design. The study protocol was approved by the leading ethics committee (Ethik-Kommission Albert-Ludwigs-Universität Freiburg) and the local ethics committees. We obtained approval and permission to conduct the study from all participating study centers. For a complete list of the committees that approved the study please refer to the Supplementary Material (Additional file 1: Table S1). The protocol was also subject to authorization by the competent authorities as mandatory by federal law. All participants have to provide written informed consent. The trial was assigned the EudraCT number 2016–001628-72 and is registered at German clinical trials registry (DRKS00011932, registration date 18 August 2017). The SPIRIT checklist of the trial is given in the Supplemental Material (see Additional file 2).

### Study objectives and endpoints

The primary objective of the study is to investigate the efficacy of age-adapted induction treatment followed by HDT-ASCT in elderly and fit patients with PCNSL. The primary endpoint of this study is 1-year PFS, where PFS is defined as time from start of treatment until disease progression or death from any cause, whichever occurs first. Secondary endpoints include rate of complete responses (CR) on day + 30 after HDT-ASCT, progression-free survival (PFS), overall survival (OS), quality of life (QoL) as measured by EORTC QLQ-C30 during therapy and 1 year after EOT [15] and non-relapse mortality (NRM). Secondary safety endpoints are (serious) adverse events, toxicity (according to NCI-CTCAE v.4.0) and neurotoxicity as measured by Mini-Mental State Examination (MMSE), EORTC QLQ-BN20 and neuro-psychological battery during therapy and 1 year after EOT [16, 17].

### Eligibility criteria

Patients must be immunocompetent, newly-diagnosed with histologically proven PCNSL of B-cell immunophenotype, aged > 65 years with an ECOG Performance Status ≤2, and eligible for HDT-ASCT as to the treating physician. For further details on inclusion and exclusion criteria please see Table 1

**Table 1:**
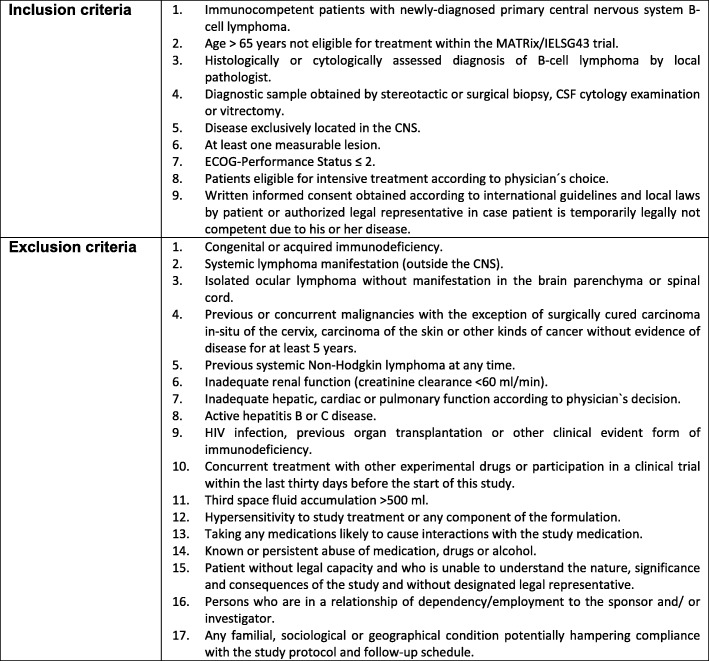
Inclusion and exlusion criteria

### Treatment schedule

**Fig. 1:**
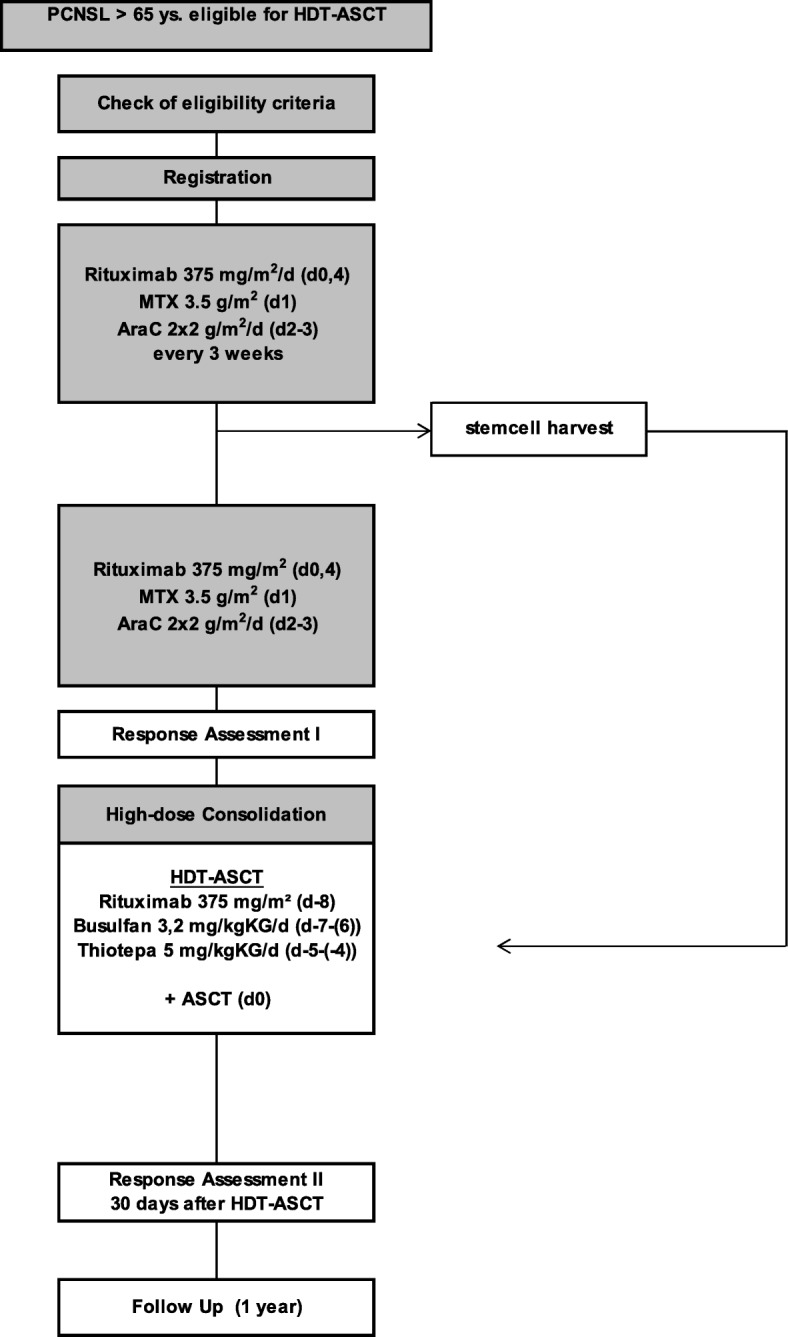
Trial Intervention Flow

The treatment schedule is summarized in Fig. 1 (Intervention Scheme).

### Induction treatment

Induction treatment consists of two cycles (every 3 weeks) of induction chemotherapy similar to the MATRix regimen [10] without thiotepa according to arm B of the IELSG32 trial [10]. Rituximab will be given intravenously at 375 mg/m^2^ on day 0 and 4 of each cycle. High-dose MTX will be administered on day 1 of each induction treatment cycle intravenously at 0.5 g/m^2^ in 15 min and then 3 g/m^2^ as a 3-h infusion. Cytarabine will be given intravenously at 2 g/m^2^ over 1 h, twice a day (every 12 h) on two consecutive days (days 2 and 3). Stem-cell harvest will be performed after the first cycle. CD34+ cells are to be collected, processed and stored according to conventional guidelines.

### Consolidation treatment

Consolidation treatment consists of HDT-ASCT with rituximab, busulfan and thiotepa: Rituximab will be given intravenously at 375 mg/m^2^ on day -8. Busulfan will be administered intravenously at 3.2 mg/kg/d over 3 h on two consecutive days (days -7 and d -6) and thiotepa will be given at 5 mg/kg over 2 h on day -5 and -4. Autologous stem cell reinfusion will be performed according to standard procedures on day 0.

### Assessments and follow up

At each visit, the following parameters will be evaluated: Eastern Cooperative Oncology Group (ECOG) Performance Status, vital signs, physical and neurological examination, laboratory profile, and adverse events, which will be graded according to NCI-CTCAE v.4.0.

Tumor response will be assessed following the IPCG response criteria [18] by an independent radiological review committee, not involved in the conception of the study.

Response assessment by brain MRI will be done after the second cycle and on day 30 after HDT-ASCT. After the end of treatment, disease status will be assessed every three months during the first year. Afterwards, control examinations are recommended to take place every six months within years 2 to 5 and annually thereafter. The trial flow chart indicating the assessments during the trial and the follow-up period is given as appendix of the SPIRIT checklist in the Supplemental Material (see Additional file 2).

Tumor size, location(s) (only at screening and in case of PD) and manifestation (singular/multiple; only at screening) will be evaluated at the aforementioned time points. In case of multiple tumors, one reference tumor will be measured, the response being evaluated by comparison to the screening MRI. Response evaluation during the trial will be determined by an independent radiological review committee not involved in the study design.

### Sample size estimation

The sample size of the trial is calculated based on the primary endpoint PFS probability at 1 year. The following assumptions are used [10, 19]: Treatment with MARTA is considered to be not successful if the 1 year PFS probability is 50% or lower. Treatment with MARTA is considered to be successful if the 1 year PFS probability is 70% or higher. The type I error rate α, i.e. the error probability of regarding the treatment regimen as successful when it is not (probability of PFS is 50% or lower), is set to 10%. The type II error rate β, i.e. the error probability of regarding the treatment regimen as not successful when it is successful (probability of PFS is 70% or higher), is set to 10% (power 90%).

For the purpose of sample size calculation, 1 year PFS is considered as a binary endpoint, and the exact binomial distribution is used to test H0: p < = 0.5 vs H1: p > =0.7 with α and β as defined above, where *p* denotes the 1 year PFS probability. The required sample size is *n* = 39, and the treatment will be considered as successful, when the number of patients who are progression free and alive after 1 year is 24 or higher.

As we assume that the disease status after one year will not be available for some patients (maximum 30%), 51 patients will be included in the trial, thus accounting for the possibility of censored observations (see next section).

### Statistical analysis

The trial will be analyzed according to the intention to treat principle (ITT), which is: All patients, for whom treatment was started, will be defined as evaluable and will be considered in the primary analysis. This set denoted as full analysis set (FAS). However, patients who withdrew consent or were removed from the study for other reasons than progression, toxicity, or death will be censored (at the date of the last radiographic assessment confirming a progression-free status).

Analysis of primary and secondary endpoints.

The primary endpoint of the trial is PFS at 1 year, where PFS is defined as the time from start of treatment until disease progression or death, whichever occurs first. The 1-year PFS probability will be estimated using the Kaplan-Meier method.

Patients will be censored at the time of last follow-up provided no event of interest has occurred so that as many patients as possible can be included in the analysis. If no censored observations before 1 year occur, the primary analysis will be conducted using the exact binomial distribution. If the number of evaluable patients progression-free and alive one year after start of treatment will be 24 or higher, treatment will be considered as successful. Two-sided confidence intervals will be derived from the exact binomial distribution for the confidence level 80% (in accordance with the specified α) and 95% (for comparability to the literature).

If censored observations occur, the 1 year PFS rate will be estimated using the Kaplan Meier method, and confidence intervals will be derived using the Greenwood formula. The treatment will be considered as successful, if the two-sided 80% confidence interval lies completely above 50%.

Endpoints of survival, where no competing risks are present (OS, PFS), will be analyzed by means of the Kaplan Meier method. In order to analyze potentially prognostic factors to a broader extend, Cox regression models are applied. Standardized questionnaires on quality of life will be analyzed descriptively in compliance with the EORTC manual.

Adverse events and serious adverse events will be registered and reported according to the guidelines of ICH/GCP. Rates of (serious) adverse events will be calculated with corresponding two-sided 95% confidence intervals. Toxicity and neurotoxicity will be analyzed descriptively. The principal coordinating investigator abstains from the option of a two-step study design with an interim analysis, because the evaluation of safety in terms of a complete toxicity assessment requires a completed study therapy. In this study the therapy lasts approximately 10 weeks.

### Quality assurance and safety

Our study data will be managed using the DAMAST Version 9.2, a proprietary data management system based on the software package SAS®, which has been developed, validated and is maintained by the Clinical Trials Unit Freiburg (CTU). SAS® software will be used to review the data for completeness, consistency and plausibility.

### Data monitoring committee

An independent Data Monitoring Committee (DMC) will be established. The DMC will consist of two medical scientists and one statistician with longstanding experience in clinical trials. The DMC’s function is to monitor the study’s course and if necessary make recommendations to the steering committee for study continuation, modification or discontinuation. The DMC will be informed about patient recruitment, adherence to the protocol, observed serious adverse events and deaths by receiving the development safety update reports (DSURs) at regular intervals. Recommendations on further continuation or modification of the study will be given to the steering committee.

## Discussion

Current treatment options for PCNSL have improved over the last years, resulting in the potential to achieve durable remission or cure in patients < 70 years. It has been shown that intensive treatment approaches including HDT-ASCT with busulfan or carmustine and thiotepa are highly effective both in first and subsequent therapy lines [5, 6, 8].

The efficacy of HDT-ASCT has been investigated in several phase II trials for primary, relapsed, or refractory PCNSL, revealing promising response and survival rates [5–7, 20]. Nevertheless, this curative treatment concept has not yet been investigated in elderly and fit PCNSL patients. The pilot study MARiTA (*N* = 15) (DRKS00008900) served the purpose of collecting data for the planning of this multicenter phase II trial and shows promising preliminary results without treatment-related mortality so far.

We now aim to answer whether age-adapted induction therapy followed by HDT-ASCT is feasible and effective in fit patients > 65 years with newly-diagnosed PCNSL in a multicenter setting. The international randomized phase II IELSG32 trial has established the combination of HD-MTX, HD-AraC, thiotepa and rituximab as the standard induction treatment for patients < 70 years eligible for HDT-ASCT [10]. To reduce toxicity (especially hematological toxicity) in elderly patients, we will use the combination of rituximab, HD-MTX and HD-AraC without thiotepa (2 cycles). In view of the highly effective HDT-ASCT protocols and with regard to the high risk of leukoencephalopathy in this special population, HDT-ASCT was chosen as consolidation. There are no conclusive prospective data regarding optimal conditioning regimen in this special population. Due to retrospective data of elderly patients with systemic lymphoma undergoing busulfan-containing HDT-ASCT, carmustine was replaced by busulfan [21]. Furthermore, as the efficacy of rituximab has been shown in the randomized phase II IELS32 trial [10], rituximab was also added as part of the consolidation therapy. Efficacy and feasibility of this combination has been shown even in patients undergoing second transplantation [22].

The study described herein follows high-level methodical and quality standards by incorporating independent pathologic and radiologic review committees and by undertaking a blinded data review after the end of the recruitment and follow-up period.

With the multicenter MARTA trial we will provide evidence regarding safety and effectiveness of an age-adapted induction treatment followed by HDT-ASCT in elderly and fit patients with newly diagnosed PCNSL.

## Additional files

Additional file 1: **Table S1.** List of study sites and ethics committees (PDF 9 kb)

Additional file 2: SPIRIT Checklist S2. SPIRIT checklist of the MARTA trial (DOC 393 kb)

## Abbreviations

(S)AE: (serious) adverse event
ASCT: Autologous stem cell transplantation
BBB: Blood brain barrier
BCNU: Carmustine
BfArM: Bundesinstitut für Arzneimittel und Medizinprodukte
CR: Complete remission
CSF: Cerebrospinal fluid
CTU: Clinical Trials Unit
d: Day
DLBCL: Diffuse large B-cell lymphoma
ECOG: Eastern Cooperative Oncology Group
EORTC QLQ-C30: European Organization for Research and Treatment of Cancer Questionnaire Core-30
HCV: Hepatitis C virus
HD-AraC: High-dose cytarabine
HD-MTX: High-dose methotrexate
HDT: High-dose chemotherapy
HIV: Human immunodeficiency virus
IELSG: International Extranodal Lymphoma Study Group
MMSE: Mini-Mental State Examination
NCI-CTCAE: National Cancer Institute - Common toxicity criteria
NHL: Non Hodgkin Lymphoma
ORR: Overall response rate
OS: Overall survival
PCNSL: Primary central nervous system lymphoma
PD: Progressive disease
PFS: Progression free survival
PR: Partial remission
QoL: Quality of life
RT: Radiotherapy
SAP: statistical analysis plan
SAS: Statistical Analysis System
SD: Stable disease
uCR: Unconfirmed complete remission
