# Supplementary material for: Age-adjusted high-dose chemotherapy and autologous stem cell transplant in elderly and fit primary CNS lymphoma patients
Source: BMC Cancer. 2019 Mar 29;19:287. doi: 10.1186/s12885-019-5473-z (PMC6440161; doi:10.1186/s12885-019-5473-z)
Supplement: Supplementary file 1 — Table S1. List of study sites and ethics committees (PDF 9 kb) [file 12885_2019_5473_MOESM1_ESM.pdf]

**MARTA Trial:**
**DRKS-No. 00011932**
**EudraCT-Number: 2016-001628-72**

# Sites and Ethic-Comittees

| | **Site of principal coordinating investigator** | | **Central ethics committee** |
|---|---|---|---|
| 1 | **Universitätsklinikum Freiburg<br>Medizinische Klinik I<br>Hämatologie/Onkologie u. Stammzelltransplantation<br>Hugstetter Str. 55<br>79106 Freiburg** | **Coordinating principal investigator<br>Dr. Elisabeth Schorb:<br>Delegate<br>Prof. Dr. Jürgen Finke** | **Ethik-Kommission<br>der Albert-Ludwigs-Universität Freiburg<br>Engelberger Straße 21<br>79106 Freiburg** |
| | **Participating sites** | **Investigators / Delegates** | **Local ethics committees:** |
| 2 | Klinikum Augsburg<br>II. Medizinische Klinik<br>Cancer Center Augsburg<br>Stenglinstr.2<br>86156 Augsburg | Investigators:<br>PD Dr. Andreas Rank<br>PD Dr. Boris Kubuschok | Ethik-Kommission der Ludwig-Maximilians Universität München<br>Pettenkoferstr. 8a<br>80336 München |
| 3 | Universitätsklinikum Essen<br>Klinik für Hämatologie<br>Hufelandstraße 55<br>45122 Essen | Investigators:<br>Prof. Dr. Alexander Röth<br>PD Dr. Andreas Hüttmann | Ethik-Kommission der Med. Fakultät der Universität Duisburg-Essen<br>Jakobs Universitätsklinikum Essen<br>Robert-Koch-Str. 9-11<br>45122 Essen |
| 4 | Klinikum der Johann-Wolfgang-Goethe-Universität<br>Medizinische Klinik II<br>Hämatologie und Onkologie<br>Theodor-Stern-Kai 7<br>60590 Frankfurt/Main | Investigators:<br>Dr. Uta Brunnberg<br>Dr. Anne Köhler | Ethik-Kommission des Fachbereichs Medizin der Johann Wolf-gang Goethe-Universität Frankfurt<br>Theodor-Stern-Kai 7<br>60590 Frankfurt am Main |
| 5 | Universitätsklinikum Göttingen<br>Abteilung<br>Hämatologie/Onkologie, Neurochirurgie<br>Robert-Koch-Str. 40<br>37075 Göttingen | Investigators:<br>Dr. med. Friederike Braulke<br>Dr. med. Justin Hasenkamp | Ethik-Kommission der Medizinischen Fakultät der<br>Georg-August-Universität<br>Von-Siebold-Straße 3<br>37075 Göttingen |
| 6 | Universitätsmedizin Greifswald<br>Klinik und Poliklinik für Innere Medizin C<br>Hämatologie, Onkologie und Transplantationszentrum<br>Sauerbruchstraße<br>17475 Greifswald | Investigators:<br>Prof. Dr. Christian A.Schmidt<br>Dr. Carsten Hirt | Ethikkommission an der Universitätsmedizin Greifswald<br>Institut für Pharmakologie<br>Felix-Hausdorff-Str.3<br>17487 Greifswald |
| 7 | Universitätskrankenhaus Hamburg-Eppendorf<br>Medizinische Klinik II<br>Onkologisches Zentrum<br>Martinistr. 52<br>20246 Hamburg | Investigators:<br>Prof. Dr. Mascha Binder<br>Dr. Winfried Alsdorf | Ethik-Kommission der Ärztekammer Hamburg<br>Weidestraße 122 b<br>22083 Hamburg |
| 8 | Universitätsklinik Heidelberg<br>Medizinische Klinik V<br>Im Neuenheimer Feld 410<br>69120 Heidelberg | Investigators:<br>Prof. Dr. Gerlinde Egerer<br>Dr. Sascha Dietrich | Ethik-Kommission I<br>der Medizinischen Fakultät Heidelberg<br>Alte Glockengießerei 11/1<br>69115 Heidelberg |
| 9 | Universitätsklinikum Köln<br>Innere Medizin 1<br>Kerpenerstr. 62<br>50937 Köln | Investigators:<br>Prof. Dr. Peter Borchmann<br>PD Dr. Bastian von Tresckow<br>Prof. Dr. Arnim Tuchscherer | Ethik-Kommission der Medizinischen Fakultät der Universität zu Köln<br>Gebäude 5, Kerpener Str.62<br>50937 Köln |

MARTA Trial:
DRKS-No. 00011932
EudraCT-Number: 2016-001628-72

# Sites and Ethic-Comittees

| | | | |
|---|---|---|---|
| 10 | Universitätsklinikum Schleswig-Holstein, Campus Kiel<br>Klinik für Innere Medizin II<br>Hämatologie und Internistische Onkologie<br>Arnold-Heller-Str. 3 (Haus 50)<br>24105 Kiel | Investigators:<br>Prof. Dr. Christiane Pott<br>Dr. Dominique Wellnitz | Ethik-Kommission der Medizinischen Fakultät der Christian-Albrechts-Universität zu Kiel<br>Arnold-Heller-Straße 3<br>Haus 9<br>24105 Kiel |
| 11 | Klinikum Stuttgart<br>Klinik für Hämatologie, Onkologie und Palliativmedizin<br>Stuttgart Cancer Center / Tumorzentrum Eva Mayr-Stihl<br>Kriegsbergstr. 60<br>70174 Stuttgart | Delegate coordinating principal investigator:<br>Prof. Dr. Gerald Illerhaus<br>Delegate:<br>Dr. Kristina Mikesch | Ethik-Kommission bei der Landesärztekammer Baden-Württemberg<br>Jahnstraße 40<br>70597 Stuttgart |
| 12 | Universitätsklinikum Tübingen<br>Medizinische Klinik II<br>Otfried-Müller-Str. 10<br>72076 Tübingen | Investigators:<br>Prof. Dr. Robert Möhle<br>Dr. Martin Sökler | Ethik-Kommission der Medizinischen Fakultät und am Universitätsklinikum Tübingen<br>Gartenstraße 47<br>72074 Tübingen |
