# Supplementary material for: Age-adjusted high-dose chemotherapy and autologous stem cell transplant in elderly and fit primary CNS lymphoma patients
Source: BMC Cancer. 2019 Mar 29;19:287. doi: 10.1186/s12885-019-5473-z (PMC6440161; doi:10.1186/s12885-019-5473-z)
Supplement: Supplementary file 2 — SPIRIT Checklist S2. SPIRIT checklist of the MARTA trial (DOC 393 kb) [file 12885_2019_5473_MOESM2_ESM.doc]

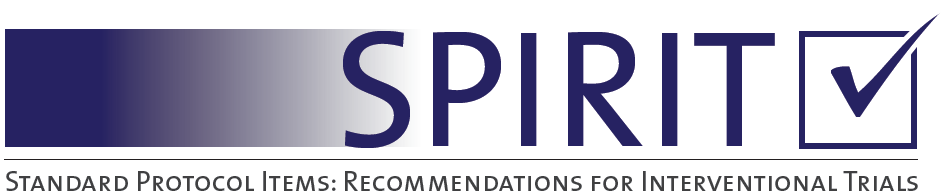


SPIRIT 2013 Checklist: Recommended items to address in a clinical trial protocol and related documents*

| Section/item | ItemNo | Description |
| --- | --- | --- |
| **Administrative information** | | |
| Title | 1 | Age-adjusted high-dose chemotherapy and autologous stem cell transplant in elderly and fit primary CNS lymphoma patients - MARTA |
| Trial registration | 2a | German clinical trials registry DRKS00011932 (registered 16 August 2017) |
|  | 2b | DRKS No.: DRKS00011932 |
| Protocol version | 3 | CTP_ V02, 02.06.2017 |
| Funding | 4 | Else Kröner-Fresenius-Stiftung |
| Roles and responsibilities | 5a | Study chairman  Dr. Elisabeth Schorb  Department of Hematology / Oncology, Freiburg University Medical Center, Freiburg, Germany  Biostatistician  Dr. rer. nat. Gabriele Ihorst  Clinical Trials Unit, Freiburg University Medical Center, Freiburg, Germany  Registration Office  Clinical Trials Unit, Freiburg University Medical Center, Freiburg, Germany  Pharmacovigilance/SAE Managment for all German sites  Clinical Trials Unit, Freiburg University Medical Center, Freiburg, Germany  Project Coordinator  Elvira Burger  Clinical Trials Unit, Freiburg University Medical Center, Freiburg, Germany  Monitoring  Heidi Fricker  Department of Hematology / Oncology, Freiburg University Medical Center, Freiburg, Germany  Data management  Clinical Trials Unit, Freiburg University Medical Center, Freiburg, Germany  Reference center radiology  Prof. Dr. Hans Henkes, Neuroradiological Medical Center, Medical Center Stuttgart, Stuttgart, Germany  Reference center pathology  Prof. Dr. Martina Deckert, University Medical Center Koeln, Institute for Neuropathology, Koeln, Germany  Data Monitoring Committee (DMC):  1. Univ. Prof. Dr. rer. nat. Geraldine Rauch (Biostatistician)  Institut fuer Medizinische Biometrie und Epidemiologie, Medical University Hospital Hamburg-Eppendorf  2. Prof. Dr. Bertram Glass (Hematologist-Oncologist)  Helios Kliniken, Klinik für Hämatologie, Onkologie und Tumorimmunologie, Berlin, Germany  3. Prof. Dr. Ulrich Herrlinger (Neurologist)  Department of Neurology - University Medical Center Bonn, Bonn, Germany |
|  | 5b | Medical Center - University of Freiburg, represented by the  Chief Medical Officer |
|  | 5c | Study Sponsor will be responsible for study design, collection management, interpretation of data, writing of the report and the decision to submit the report for publication. Sponsor has ultimate authority over any of these activities. |
|  | 5d | Composition, roles, and responsibilities of the coordinating centre, endpoint adjudication committee, data management team, and other individuals or groups overseeing the trial, not applicable see Item 21a for data safety monitoring committee. |
| Introduction |  |  |
| Background and rationale | 6a | Primary central nervous system lymphoma (PCNSL) is an aggressive Non-Hodgkin Lymphoma (NHL) mostly of B-cell origin, which exclusively invades the central nervous system compartment. It accounts for 3% to 4% of all primary brain tumours and 4% to 6% of extra-nodal lymphomas [1]. The incidence of PCNSL in immunocompetent patients has been steadily increasing over the last 30 years [2, 3]. High-dose methotrexate (HD-MTX) in combination with HD-cytarabine (HD-AraC) is the backbone of current treatment [4]. A recent randomized controlled trial investigated the role of whole brain radiotherapy (WBRT) as consolidation therapy compared to no consolidation therapy, suggesting that WBRT does not prolong survival but enhances disease control [5]. However, despite treatment improvement, the prognosis of PCNSL patients is still poor compared to systemic Non-Hodgkin Lymphoma [6].  High-dose chemotherapy followed by ASCT is known to be a highly effective treatment strategy for NHL. In the pre-rituximab-era it was the therapeutic backbone for the treatment of relapsed and refractory diffuse large B-cell lymphoma (DLBCL) as well as first-line treatment for high-risk DLBCL. Treatment of PCNSL differs from that in other DLBCL-locations due to the fact that most of the active drugs in NHL-treatment cannot pass the blood-brain barrier (BBB). This problem can be overcome by using drugs penetrating the CNS and/or by increasing the doses in order to reach higher drug-levels in the CNS.  The most active chemotherapeutic drug in the treatment of PCNSL is MTX with doses higher than 1,5g/m2. Beside protocols applying HD-MTX as a single agent, various treatment regimens containing additional chemotherapeutics have been proposed. However, the only agent yet tested in a randomized trial is cytarabine, which - in addition to HD-MTX - leads to improved response rates. [18] Based on experience with other hematologic malignancies and the need for effective consolidation treatment, HDT-ASCT was also evaluated in PCNSL. The rationale for the impact of HDT-ASCT in PCNSL is its delivery into the CNS of BBB penetrating agents at several-fold higher concentrations than conventional therapy, which cannot provide such penetration. [19, 20] In recent trials we demonstrated a high rate of continuous remissions after treatment with HDT-ASCT with or without WBRT. In a first pilot and phase-II study, we treated 30 patients with PCNSL ≤ 65 years with sequential induction chemotherapy including three cycles of HD-MTX, HD-AraC, and thiotepa followed by stem-cell harvest. The conditioning regimen consisted of carmustine and thiotepa followed by ASCT; WBRT was given as consolidation. [11] Twenty-three of 30 patients proceeded to HDT-ASCT resulting in CR and PR in 15 and 8 patients, respectively. Twenty-one patients subsequently underwent WBRT and all achieved CR. With a median follow-up of 63 months, the 5-year OS was 69% for all patients and 87% for those completing HDT and ASCT, respectively. In a follow-up pilot study induction chemotherapy was intensified by doubling the thiotepa dose to restrict WBRT to those not achieving CR. [12] Seven of eleven patients were in CR following ASCT and 3 in PR upon ASCT received radiotherapy as consolidative treatment. After a median follow-up of 25 months, 3-year OS was 77%. None of the patients suffered from severe neurotoxicity during the follow-up period. Both trials suggest a curative effect of HDT-ASCT in young PCNSL patients. This concept supplemented by rituximab was evaluated in a multicentre phase II trial including 79 patients ("Freiburg ZNS-NHL Trial", ClinicalTrials.gov Identifier: NCT00647049). Overall remission rate (ORR) was 91% (77% CR and 14% PR). After a median follow-up of 35 months, 3-year overall survival was 77.6% for all patients and 87.1% for patients after HDT- ASCT. In light of these positive findings, we have initiated an ongoing international randomized phase-II trial (2 randomizations, 1st 3 arms, 2nd 2 arms) in collaboration with the International Extranodal Lymphoma Study Group (IELSG) on primary chemotherapy with HD-MTX and HD-AraC with or without thiotepa and with or without rituximab (1st randomization), followed by whole brain radiotherapy vs. high-dose chemotherapy supported by autologous stem-cell transplantation (2nd randomization) for immunocompetent patients with newly-diagnosed primary CNS lymphoma (ClinicalTrials.gov Identifier: NCT01011920). This trial provides a high level of evidence supporting the use of the MATRix combination as the new standard chemoimmunotherapy for PCNSL patients up to 70 years [21]. In a few months we will also be able to determine the superiority of HDT-ASCT or WBRT as consolidation treatment.  In the ongoing MATRix/IELSG43 Trial, a randomized, phase III trial in collaboration with the International Extranodal Lymhoma Study Group (IELSG), the efficacy measured as progression- free survival (PFS) of intensive chemotherapy followed by autologous stem-cell transplantation is compared to conventional chemotherapy (ClinicalTrials.gov Identifier NCT02531841) [22].  This multicentre trial is being conducted in 35 centres in Germany  and 45 international centres of the IELSG. Elisabeth Schorb is the  medical coordinator of this trial. Gerald Illerhaus is the Principal Coordinating Investigator and Jürgen Finke is the Deputy Coordinating Principal Investigator of the German centres. The "Freiburg Protocol", the "IELSG32 Trial" and the "MATRix/IELSG43 Trial" are conducted by the "Cooperative Primary CNS-Lymphoma Study Group" with 35 medical centres in Germany. In patients >60 years of age, WBRT in combination with MTX-based chemotherapy causes high rates of leukoencephalopathy with dementia, ataxia, gait disturbances, and incontinence, all significantly decreasing quality of life in surviving patients. [23, 24] There have been very few studies using MTX-based protocols without WBRT focusing on elderly PCNSL patients [25-27]. Our study group introduced the MCP regimen consisting of HD-MTX (3 g/m2) in combination with the oral lipophilic alkylating agents lomustine and procarbazine for patients >65 years of age, restricting WBRT to those not responding to chemotherapy [28]. In a subsequent single centre study, we added rituximab to the MCP protocol which achieved 1- and 3-year OS rates of 67.9% (95% CI 47.3% to 81.8%) and 31.1% (95% CI 14.2% to 50%), respectively [29]. This protocol has been tested in a multicentre setting (PRIMAIN study) with similar survival rates compared to the single centre experience (manuscript under review at "Leukemia"). |
|  | 6b | Explanation for choice of comparators  The current standard recommendation for previously untreated PCNSL is the combination of HD-MTX and HD-AraC followed by WBRT [4] In order to improve the results in patients < 65-70 years the combination of HD-MTX/AraC with thiotepa and rituximab was investigated in consecutive trials demonstrating feasibility and high efficacy. In order to reduce toxicity in elderly patients we chose the combination of rituximab, HD-MTX and HD-AraC without thiotepa (2 cycles). In view of the high efficacy of the Freiburg-Protocol HDT-ASCT was chosen as consolidation, but carmustine was replaced by busulfan in order to reduce toxicity in this special population. Furthermore, as the efficacy of rituximab has been shown in the randomized phase II IELS32 trial [21], rituximab was also added as part of the consolidation therapy. Efficacy and feasibility of this combination has been shown even in patients undergoing second transplantation [30]. |
| Objectives | 7 | The primary objective of the study is to investigate the efficacy of age-adapted induction treatment followed by high-dose chemotherapy and autologous stem cell transplantation regarding 1-year PFS in elderly and fit patients with primary CNS lymphoma. Secondary objectives are the investigation of OS, treatment response (rate of complete responses on day 30 after HDT-ASCT) and treatment related morbidities (neurotoxicity and adverse advents). |
| Trial design | 8 | This is an open-label, prospective, multicentric, non-randomized, single arm phase II trial using Fleming one stage design. |
| Study setting | 9 | We recruit patients from 12 hospitals in Germany. If necessary, additional qualified sites can be included during the course of the trial. For a list of the currently recruiting centers in Germany please refer to the Appendix section. |
| Eligibility criteria | 10 | Inclusion criteria:  1. Immunocompetent patients with newly-diagnosed primary central nervous system B-cell lymphoma.  2. Age > 65 years not eligible for treatment within the MATRix/IELSG43 trial.  3. Histologically or cytologically assessed diagnosis of B-cell lymphoma by local pathologist.  4. Diagnostic sample obtained by stereotactic or surgical biopsy, CSF cytology examination or vitrectomy.  5. Disease exclusively located in the CNS.  6. At least one measurable lesion.  7. ECOG-Performance Status ≤ 2.  8**.** Patients eligible for intensive treatment according to physician´s choice.  9. Written informed consent obtained according to international guidelines and local laws by patient or authorized legal representative in case patient is temporarily legally not competent due to his or her disease.  Exclusion criteria:  1. Congenital or acquired immunodeficiency.  2. Systemic lymphoma manifestation (outside the CNS).  3. Isolated ocular lymphoma without manifestation in the brain parenchyma or spinal cord.  4. Previous or concurrent malignancies with the exception of surgically cured carcinoma in-situ of the cervix, carcinoma of the skin or other kinds of cancer without evidence of disease for at least 5 years.  5. Previous systemic Non-Hodgkin lymphoma at any time.  6. Inadequate renal function (creatinine clearance <60 ml/min).  7. Inadequate hepatic, cardiac or pulmonary function according to physician`s decision.  8. Active hepatitis B or C disease.  9. HIV infection, previous organ transplantation or other clinical evident form of immunodeficiency.  10. Concurrent treatment with other experimental drugs or participation in a clinical trial within the last thirty days before the start of this study.  11. Third space fluid accumulation >500 ml.  12. Hypersensitivity to study treatment or any component of the formulation.  13. Taking any medications likely to cause interactions with the study medication.  14. Known or persistent abuse of medication, drugs or alcohol.  15. Patient without legal capacity and who is unable to understand the nature, significance and consequences of the study and without designated legal representative.  16. Persons who are in a relationship of dependency/employment to the sponsor and/ or investigator.  17. Any familial, sociological or geographical condition potentially hampering compliance with the study protocol and follow-up schedule. |
| Interventions | 11a | All enrolled patients will receive induction chemotherapy with rituximab, HD-MTX and HD-Ara-C. The maximum number of induction chemotherapy courses will be two. Chemotherapy will be administered every three weeks. Patients in complete remission (CR), unconfirmed complete remission (CRu), partial remission (PR) or stable disease (SD) after one course will receive additional course of the primary chemotherapy regimen. Stem-cell harvest will be performed after the first course for all patients.  Response assessment by brain MRI will be done after the second course. Patients who experience progressive disease (PD) at any time will be assigned to off-study salvage therapy. Patients whose stem-cell harvest is insufficient after two cycles of induction treatment will be treated off-study as well.  Patients achieving SD, PR or CR after 2 cycles of induction chemotherapy will undergo consolidating high-dose chemotherapy with busulfan and thiotepa followed by ASCT.  After the end of treatment visit, patients will remain in follow-up for 12 months. Further annual control examinations are recommended. Patients who achieve a CR, unconfirmed CR or PR (in comparison to baseline MRI) will proceed to regular follow-up. In case of CRu and PR (in comparison to baseline MRI), close monitoring of the patient by MRI is recommended to confirm the response status. Patients with PD (in comparison to baseline / best response) or relapse after end of study treatment will undergo WBRT or second high dose chemotherapy according to the investigators choice. |
|  | 11b | Discontinuation criteria for the individual patient: In case of PD at any time, insufficient stem-cell harvest, inadequate bone marrow recovery (discontinuation of chemotherapy in case of delay of more than four weeks) trial treatment is terminated prematurely.  Investigators should follow the guidelines below for dose modification of treatment with investigational product; any deviation must be discussed previously with the sponsor unless it concerns a patient’s safety. All dose changes or interruptions must be recorded on the appropriate CRF page.  In case of inadequate bone marrow recovery, that is ANC <1.500/μl (<1.200/μl in arm “A”) and platelets <90.000/μl, on the intended day of re-treatment, the start of the next cycle can be delayed for a maximum of 4 weeks. Thereafter, chemotherapy must be discontinued, and patients will be treated according to the investigator's decision.  In case of non-hematologic toxicity dose modification should be managed according to the investigator`s judgement and as recommended in the drug information. Rituximab infusion reactions will be managed according to international guidelines. |
|  | 11c | Strategies to improve adherence to intervention protocols, and any procedures for monitoring adherence (eg, drug tablet return, laboratory tests)  Information about drugs given to the patient must be entered in the source documents and in the CRFs to accurately determine the patient’s drug exposure throughout the trial.  Drug accountability will be performed according to the routine pharmacy practice without study specific documentation in the Investigator Site/Pharmacy File. |
|  | 11d | Permitted medication: Patients with newly-diagnosed PCNSL often receive corticosteroids to reduce perifocal brain edema. However, whenever possible, steroids should be tapered out as early as possible before biopsy or at the very latest, once chemotherapy has started. If patients suffer from epilepsy due to lymphoma manifestations, anti-epileptic drugs are allowed and can be continued as clinically indicated. The patient must notify the investigational site about any new medications he or she is taking after starting the trial medication. The following drugs can be applied in our trial: antiemetics, analgesics, antibiotics, anticonvulsants, sedatives, antihyperuricemic agents as well as other therapies to control metabolic and malnutrition disturbances. Corticosteroids during treatment and their definitive interruption will depend on clinical requirements. It is important that the type and doses of corticosteroids be accurately recorded. Oral antiviral, antifungal and antipneumocystic prophylaxis is strongly suggested. Antimicrobial drugs should be interrupted during chemotherapy administration to avoid potentially negative pharmacological interactions. Rituximab infusion reactions will be managed according to international guidelines. Folinic-acid rescue starts 24 hours after the start of MTX infusion and is recommended to be scheduled and documented according to the attached leucovorin rescue sheet. The post-MTX hydration should attain a total volume of 2000 ml. All further medications during the trial must be documented in the patient file, as well as the indication, dosage and period of administration. All medications not triggering known interactions can be given during the trial if necessary and clinically indicated. Corticosteroids during treatment and their definitive interruption will depend on clinical requirements. Oral antiviral, antifungal and antipneumocystic prophylaxis is strongly suggested. Antimicrobial drugs should be interrupted during chemotherapy administration to avoid potentially negative pharmacological interactions. The post-MTX hydration should attain a total volume of 2000 ml. All further medications during the trial must be documented in the patient file, as well as the indication, dosage and period of administration. All medications not triggering known interactions can be given during the trial if necessary and clinically indicated.  Additional cytotoxic therapy, biological responsive modifiers and drugs possibly interfering in the action or pharmacokinetics of rituximab, MTX, Ara-C, thiotepa, or busulfan must be avoided. |
| Outcomes | 12 | The primary endpoint of the trial is PFS at 1 year, where PFS is defined as the time from start of treatment until disease progression or death, whichever occurs first. The 1-year PFS probability will be estimated using the Kaplan-Meier method.  Secondary objectives are  1. Rate of complete responses (CR) on day +30 after HDT-ASCT  2. Progression-free survival (PFS) as time from start of treatment until progression, relapse or death from any cause, whatever happens first  3. Overall survival (OS) as time from start of treatment until death from any cause  4. Rate of neurotoxicity on day + 30 after HDT-ASCT and continuously thereafter  5. Non-relapse mortality (NRM) will be determined from time to death from other causes than relapse or disease progression, considering death from relapse or disease progression as a  competing event  6. (Serious) adverse events ([S]AEs): from the first administration of the study medication until day 30 after HDT-ASCT. |
| Participant timeline | 13 | Please refer to the Appendix Section (Study Flow Chart) |
| Sample size | 14 | The sample size of the trial is calculated based on the primary endpoint PFS probability at 1 year. The following assumptions are used [21, 29]: Treatment with MARTA is considered to be not successful if the 1 year PFS probability is 50% or lower [21].Treatment with MARTA is considered to be successful if the 1 year PFS probability is 70% or higher.  - The type I error rate α, i.e. the error probability of regarding  the treatment regimen as successful when it is not (probability of  PFS is 50% or lower), is set to 10%.  - The type II error rate β, i.e. the error probability of regarding the  treatment regimen as not successful when it is successful (probability  of PFS is 70% or higher), is set to 10%.  For the purpose of sample size calculation, 1 year PFS is considered as a binary endpoint, and the exact binomial distribution is used to test H0: p<= 0.5 vs H1: p>=0.7 with α and β as defined above, where p denotes the 1 year PFS probability. The required sample size is n=39, and the treatment will be considered as successful, when the number of patients who are progression free and alive after 1 year is 24 or higher. As we assume that the disease status after one year will not be available for a few patients (maximum 30%), 51 patients will be included in the trial. |
| Recruitment | 15 | 12 leading German hematological and neuro-oncological centers are participating in the trial. |
| **Methods: Assignment of interventions (for controlled trials)** | | |
| Allocation: |  |  |
| Sequence generation | 16a | not applicable |
| Allocation concealment mechanism | 16b | not applicable |
| Implementation | 16c | not applicable |
| Blinding (masking) | 17 | not applicable |
| **Methods: Data collection, management, and analysis** | | |
| Data collection methods | 18a | Physical and neurological examinations are recommended to be done according to the flow chart; detailed findings concerning neurological examinations must be documented in the CRF at screening. Hematological tests and blood chemistry should be done twice a week during therapy. Data on physical examination, vital signs, and neurological evaluation will be collected by means of toxicity tables before initiating each chemotherapy cycle and at the EOT visit.  Laboratory data will be documented at screening, during therapy, at the EOT visit and during follow-up as toxicity parameters and graded according to CTCAE 4.0. Hematology includes assessment of white blood count (WBC), neutrophils, hemoglobin, and platelets. Blood chemistry comprises creatinine, total bilirubin, ALT, AST, and gamma-GT (only at screening).  During therapy creatinine must be documented in the CRF in the measured unit to allow approximation of the kidney glomerular filtration rate (GFR) using the MDRD (Modification of Diet in Renal Disease) formula. Lactate dehydrogenase will be documented at screening by indicating "not increased" or "increased".  If a symptom/diagnosis/laboratory parameter is not available on the toxicity table, an AE must be documented on the CRF AE page. If any serious criterion is fulfilled, an SAE must be reported. |
|  | 18b | Screening failures are defined as patients who signed an ICF but failed to be registered in the study for any reason. These patients are to be documented on the subject screening log (see section 6.1). For these patients, the screening CRFs and CRF pages with inclusion/exclusion criteria must be completed and "screening failure" stated on the registration fax naming at least one inclusion criteria not fulfilled or one exclusion criteria present. |
| Data management | 19 | All data collected during the trial for each patient registered will be entered on the trial-specific electronic CRF pages by the responsible investigator or an individual who is designated by the investigator as soon as possible. The investigator is responsible for ensuring the accuracy completeness, and timeliness of the data reported to the sponsor in the eCRFs and in all required reports.  In order to identify authorized study personnel, a signature log is to be kept in the ISF and is to be forwarded to the sponsor for archival in the TMF. Accurate and reliable data collection will be assured by verification and cross–check of the eCRFs against the investigator’s records by the study monitor (source data verification). The investigator ensures that the participation of the patients in the study is documented in the patient’s hospital records. At each trial center a separate patient identification list needs to be kept updated and filed in the ISF. All data collected during the trial will be entered on the trial-specific eCRF pages by the responsible investigator, or a person designated by the investigator. Corrections and subsequent changes to CRF pages must be made according to the ICH-GCP guidelines provided in the eCRF completion instructions.  The study data will be managed using the DAMAST Version 9.2, a proprietary data management system based on the software package SAS®, which has been developed, validated and is maintained by the Clinical Trials Unit (CTU). Details on data management (procedures, responsibilities, data corrections, for which data management staff of the CTU is responsible) will be described in a data management manual (DMM) prior to the trial. The data management manual is a working document and also contains a record of all data management processes carried out during the clinical trial. Before any data is entered, the trial database will be validated and specifications of the database will be documented in a variables handling plan. An audit trail will be created to provide an electronic record of which data were entered or subsequently changed by whom and when.  SAS® software will be used to review the data for completeness, consistency and plausibility. The checks to be programmed will be specified beforehand in a data validation plan. After running the check programs, the resulting queries will be sent to the investigator for review of his/her data. Answered queries will also be entered twice, verified and the updated data will then be transferred to the database. All programs which can be used to influence the data or the data quality will be validated (e.g. check programs, programs used for the input of external data, etc.). |
| Statistical methods | 20a | Primary endpoint: The primary endpoint of the trial is PFS at 1 year, where PFS is defined as the time from start of treatment until disease progression or death, whichever occurs first. Patients with observation times below one year will be censored (at the date of the last radiographic assessment confirming a progression-free status). Reasons for censored observations where no assessment of disease status after one year is possible include trial termination due to toxicity, withdrawal of informed consent, or other reasons. If no censored observations before 1 year occur, the primary analysis will be conducted using the exact binomial distribution. If the number of evaluable patients progression-free and alive one year after start of treatment is 42 or higher, treatment will be considered as successful. Two- sided confidence intervals will be derived from the exact binomial distribution for the confidence level 80% (in accordance with the specified α) and 95% (for comparability to the literature). If censored observations occur, the 1 year PFS rate will be estimated using the Kaplan Meier method, and confidence intervals will be derived using the Greenwood formula. The treatment will be considered as successful, if the two-sided 80% confidence interval lies completely above 50%.  Secondary endpoints: Survival endpoints where no competing risks exist (OS, PFS) will be analyzed by means of the Kaplan Meier method. Descriptive analysis of secondary endpoints will be performed with two- sided 95% confidence intervals. In case competing events are present (e.g. NRM), estimation via cumulative incidence rates using the Aalen Johansen estimator will be applied. Standardized questionnaires on quality of life will be analyzed descriptively in compliance with the EORTC manual. CR rate on day 30 after HD-ASCT will be calculated based on all patients included in the FAS. Patients with missing response assessment due to death or premature study termination will be evaluated as non-responders. Quality of life (QOL) of patients is evaluated using the EORTC QLQ-C30 and EORTC QLQ- BN20 Quality of Life questionnaire. The questionnaire is answered by the patients at screening, the EOT visit and one year after EOT. For evaluating QOL, scores will be calculated according to the EORTC manual. [45] The scores at screening will be summarized and for different time points during follow-up, differences of the scores to the screening scores will be calculated. |
|  | 20b | In order to analyze potentially prognostic factors to a broader extend, Cox regression models will be applied. |
|  | 20c | The trial will be analysed according to the intention to treat principle. Therefore, all patients, for whom treatment was started, will be defined as evaluable and will be considered in the primary analysis. This set denoted as full analysis set (FAS). However, patients who withdrew consent or were removed from the study for other reasons than progression, toxicity, or death will be censored (at the date of the last radiographic assessment confirming a progression-free status). |
| **Methods: Monitoring** | | |
| Data monitoring | 21a | An independent Data Monitoring Committee (DMC) will be established. The DMC consists of two medical scientists and one statistician with longstanding experience in clinical trials (see section Responsibility page 22). The DMC’s function is to monitor the study’s course and if necessary make recommendations to the steering committee for study discontinuation, modification or continuation. The underlying principles for the DMC are the patients’ ethical and safety aspects. It is the task of the DMC to examine whether the study’s conduct is still ethically justifiable, whether security of the patients is ensured and whether the study’s conduct is acceptable. The DMC will be informed about adherence to the protocol, patient recruitment, observed serious adverse events and deaths. The DMC will receive the corresponding reports (DSURs) at regular intervals. Recommendations on further continuation or modification of the study will be given to the steering committee. The composition and responsibilities of the DMC, the structure and procedures of its meetings, and its relationship to other key study team members (steering committee), will be laid down in a separate DMC charter. |
|  | 21b | The principal coordinating investigator abstaines from the option of a two-step study design with an interim analysis, because the evaluation of safety in terms of a complete toxicity assessment requires a completed study therapy. In this study the therapy lasts approximately 10 weeks.  The clinical trial must be terminated prematurely if:  - the benefit-to-risk ratio for the patient changes markedly,  - the sponsor/principal coordinating investigator (German LKP) or the DMC considers that the termination of the trial is necessary,  - indications arise that the trial patients' safety is no longer guaranteed,  - questions, addressed in the trial, can be clearly answered on the basis of results from another trial on the same subjects.  If the clinical trial is prematurely terminated or suspended for any reason, the investigator should promptly inform the trial patients and ensure their appropriate therapy and follow-up. Where required by the applicable regulatory requirements, the competent authority(ies) and the ethic committee(s) will also be informed. This is done by the sponsor. |
| Harms | 22 | An adverse event (AE) is any untoward medical occurrence in a patient administered any dose of a pharmaceutical product and which does not necessarily have to have a causal relationship with the product’s use. An AE can therefore be any unfavourable and unintended sign (including an abnormal laboratory finding), symptom, or disease temporally associated with the use of an investigational medicinal product, whether or not related to the product. Irrespective of any causal relationship, all AEs spontaneously reported by the patient or observed by the investigator will be continuously documented in the medical record and on the designated case report form (AE CRF page). All AEs must be described by diagnosis or, if an underlying diagnosis is not known, by symptoms or medically significant laboratory or instrumental abnormalities. The AEs will be documented as shown in the section below. Symptoms or medically-significant laboratory or instrumental (e.g. electro-cardiographic) abnormalities concerning a pre-existing disease should not be considered an AE. However, occurrences of new symptoms or laboratory or instrumental abnormalities as well as worsening of pre-existing symptoms are considered AEs. In order to monitor the conditions of the patients from the point in time when they receive the first dose of the investigational product, the investigator is requested to report any untoward clinical event on an AE-page of the CRF. Any untoward medical occurrence that occurs after the patient’s follow-up period as defined in the protocol is not considered an AE. All AEs, no matter how intense, are to be followed up by the investigator in accordance with good clinical practice until resolved or judged no longer clinically relevant, or in case of a chronic condition, until it is fully characterized. Adverse events must be documented in the CRF starting from the first administration of the study medication until day 30 after HDT-ASCT. Afterwards, only serious AEs judged by the investigator to be related to at least one of investigational products (see section 2.3) have to be documented on the AE-page in the CRF and reported to the sponsor accordingly. During induction and consolidation treatment and before beginning each chemotherapy cycle, the CRF-toxicity table must be thoroughly completed by the investigator. The investigator has to state the presence of a toxicity grade (CTCAE) in terms of several laboratory parameters and symptoms or diagnoses. If the symptom, diagnosis, or laboratory parameter is not stated on the toxicity table, an AE must be documented on the CRF AE page. If any of the seriousness criteria are fulfilled, an SAE must be reported.  All SAEs, that occur starting from the first administration of the study medication until day 30 after HDT-ASCT, will be documented in the CRF and on the provided SAE reporting form. This applies to events, that are only documented on the toxicity table, that are **related and not related** to the study medications, and of any CTCAE grade. After this time period, an SAE need only be reported to the sponsor and documented on the CRF if judged by the investigator to be related to at least one of investigational products.  All SAEs must be reported by fax within 24 hours after knowledge by the investigator. According to section 4, § 12, subsection 6 GCP-V, in the event of the death of a patient, the investigator must submit all information to the competent ethics committee, the other ethics committee involved, and the competent authority and sponsor that is required for the fulfillment of his or her duties (note that personal data must be transmitted using the trial-specific patient identification number, i.e. in anonymized form).  An exception to this principal rule is: As this trial involves patients suffering from malignancies associated with significant mortality/morbidity, and because relapse/progression are trial endpoints (i.e. anticipated clinical outcomes) collected on the specific CRF pages and taking into consideration recommendations of the CIOMS working group VI concerning management of safety information from clinical trials, the investigator does not need to inform the sponsor about the events below, as they are not considered SAEs in this disease context: relapse, progression, patient’s death.  Please note that “death” is usually an SAE outcome and not an SAE per se. Only in cases where the clinical circumstances before death are unknown (i.e. patient died without a definable cause of death), the diagnosis “death” itself should be reported as an SAE. In case of fatal outcome of an already reported SAE a follow-up notification must be sent.  The sponsor's expedited reporting requirements are particularly relevant to suspected unexpected serious adverse reactions (SUSARs). The definition is a combination of the definitions of serious adverse reaction (for seriousness criteria see section 11.1.3) and unexpected adverse reaction (an adverse reaction, the nature or severity of which is inconsistent with the applicable product information for the investigational medicinal product). The sponsor's reporting requirements are divided into **expedited** reporting and reporting that must be done annually or on request. The sponsor's expedited reporting requirements comprise the following:  All SUSARs must be reported within 15 days after knowledge (§ 13, subsection 2 GCP- V). All SUSARs that are life-threatening or result in death must be reported within 7 days after knowledge (§ 13, subsection 3 GCP-V). All circumstances requiring a review of the benefit/risk evaluation of the investigational medicinal product must be reported within 15 days after knowledge (e.g. expected serious adverse reaction with unexpected outcome, increased incidence of expected serious adverse reactions, SUSARs after the end of the patient's participation in the clinical trial, events in connection with the trial conduct or the development of the investigational medicinal product which may affect the safety of the trial patients) (§ 13, subsection 4 GCP-V).  Development Safety Update Report (DSUR):  In addition to the expedited reporting, the sponsor shall submit an annual report **once a year** or on request throughout the clinical trial period, according to § 13, subsection 6 GCP-V and ICH guideline E2F. The aim of the DSUR is to concisely describe all new safety information relevant for one or several clinical trial(s) and to assess the safety conditions of subjects included in the trial(s) concerned.  Any pregnancy (female trial participant or partner of male trial participant) that occurs during trial participation must be reported to the Sponsor on the pregnancy reporting form within 24 hours of learning of its occurrence. The pregnancy should be followed up by the sponsor to determine outcome, including spontaneous or voluntary termination, details of birth, and the presence or absence of any birth defects, congenital abnormalities or maternal and newborn complications. |
| Auditing | 23 | According to the ICH-GCP guidelines audits will be performed within a quality assurance system. Audits and/or inspections may be conducted by the sponsor, authority(ies) or an independent external party. The purpose of a sponsor’s audit, which is independent of and separate from routine monitoring or quality control functions, should be to evaluate trial conduct and compliance with the protocol, SOPs, GCP, and the applicable regulatory requirements. All persons who conduct an audit undertake in writing to treat all data related to medical secrecy or which could reveal the patient's identity in absolute confidence and to restrict the use of such data to the purposes agreed by the patient in writing.  Proposed dates for sponsor’s audit, characteristics of the selected patients and further information will be transmitted to the investigator by the CRA in a timely manner.  The investigator will inform the CTU immediately of an inspection requested by a regulatory authority. The investigator is responsible for making source data/documents available during audits or inspections. |
| Ethics and dissemination | | |
| Research ethics approval | 24 | not applicable |
| Protocol amendments | 25 | Any change or addition to the protocol can only be made in a written protocol amendment that must be approved by sponsor, CA where required, and the IEC.  Only changes to the protocol that are required for patient safety may be implemented prior to IEC approval. Despite the need for approval for formal protocol amendments, the investigator is expected to take any immediate action required to ensure the safety of any patient enrolled in this trial, even if such action represents a protocol deviation. In such cases, the sponsor should be notified as soon as possible of this action; the IEC should be informed correspondingly. |
| Consent or assent | 26a | Before enrolment in the clinical trial, the patient will be informed that participation in the clinical trial is voluntary and that he/she may withdraw from the clinical trial at any time without having to give reasons and without penalty or loss of benefits to which the patient is otherwise entitled. The treating physician will provide the patient with information about the treatment methods to be compared and the possible risks involved. At the same time, the nature, significance, implications, expected benefits and potential risks of the clinical trial and alternative treatments will be explained to the patient. During the informed consent discussion, the patient will also be informed about the insurance cover that exists and the insured's obligations. The patient will be given ample time and opportunity to obtain answers to any open questions. All questions relating to the clinical trial should be answered to the satisfaction of the patient and/or his/her legal representative. In addition, the patient will be given a patient information sheet which contains all the important information in writing. By signing the consent form, the patient agrees to voluntarily participate in the clinical trial and declares his/her intention to comply with the requirements of the clinical trial and the investigator's instructions during the clinical trial. By signing the form, the patient also declares that he/she agrees to the recording of personal data, particularly medical data, for the trial, to their storage and codified (“pseudonymized”) transmission to the sponsor, to the ethics committee or the competent authority, and further agrees that authorized representatives of the sponsor, the Medical Center – University of Freiburg, who are bound to confidentiality, and national or foreign competent authorities may inspect his/her personal data, particularly medical data, which are held by the investigator. After having signed the informed consent, the patient will be given one copy of the signed and dated written consent form and any other written information to be provided to the patients. In the case of substantial amendments, the patient must be informed with an appropriate revised patient information/consent form. Changed trial procedures can only be carried out if they have been approved by the competent authority and the leading ethics committee, and if the patient has been appropriately informed and has given his/her written consent. Fertile men and women of child-bearing potential should be informed that taking the investigational product may involve unknown risks to the fetus if pregnancy were to occur during the trial and agree that in order to participate in the trial, they must adhere to the contraception requirement for the trial’s duration. If there is any doubt that the patient may not reliably comply, they should not be registered for the trial. |
|  | 26b | not applicable |
| Confidentiality | 27 | Information about trial patients will be kept confidential and managed under the applicable laws and regulations. Those regulations require a signed patient authorization informing the patient of the following: what protected health information (PHI) will be collected from patients in this trial; who will have access to that information and why; who will use or disclose that information; Right of trial patients to revoke their authorization for use of their PHI.  In the event that a patient revokes authorization to collect or use PHI, the investigator, by regulation, retains the ability to use all information collected prior to the revocation of patient authorization. For patients who have revoked authorization to collect or use their PHI, attempts should be made to obtain permission to collect at their least vital status (i.e. that the patient is alive) at the end of their scheduled trial phase.  For RDE light-based trials (using DAMAST, SAS®-based data management system):  The data collection system for this trial uses built-in security features to prevent unauthorized access to confidential participant information. Access to the system will be controlled by individually-assigned user identification codes and passwords made available only to authorized personnel who have completed prerequisite training. The investigator must ensure anonymity of the patients; patients must not be identified by names in any documents submitted to the sponsor. Signed informed consent forms and patient enrolment log must be kept strictly confidential to enable patient identification at the site. |
| Declaration of interests | 28 | ES, JF and GI receive speakers honoraries from Riemser Pharma GmbH. All other investigators declare that they have no competing interests. |
| Access to data | 29 | Sponsor and study chairman (coordinating principal investigator) is responsible for access to data set. This item is stipulated in details in the site contracts between sponsor, chairman (coordinating PI) and participating sites. |
| Ancillary and post-trial care | 30 | The treatment and care of patients after the end of study is conducted according to oncological and haematological standards in the respective sites.  Subject insurance (minimum: € 500,000 per subject) for all subjects participating in the clinical trial for compensation to those who suffer harm from trial participation. |
| Dissemination policy | 31a | Upon trial completion, the results of this trial will be submitted for publication and/or posted in a publicly-accessible database of clinical trial results. Reporting guidelines will be taken into account (see www.equator-network.org), e.g. the CONSORT statement should be adhered to when drafting papers on the results of randomized studies.  Each publication of trial results will occur in mutual agreement between the principal investigator, any other investigators involved, and the CTU. All data collected in connection with the clinical trial will be treated in confidence by the coordinating investigator and all others involved in the trial until publication. Interim data and final results may only be published (orally or in writing) with agreement from the coordinating investigator and the CTU. This is essential for a thorough exchange of information between the afore mentioned parties and will ensure that the opinions of all parties involved have been heard before publication. This agreement, which does not include any veto right or right of censorship for any of the parties involved, may not be refused without good reason. |
|  | 31b | Study chairman (coordinating principal investigator) is responsible for publication. Details with reference to publication rights are stipulated in the site contracts between sponsor, chairman (coordinating PI) and participating sites.  Any intended use of professional writers: not applicable |
|  | 31c | Plans, if any, for granting public access to the full protocol, participant-level dataset, and statistical code: not applicable |

**Appendices**

Literature

Flow Chart

**Literature**

1. Nelson DF. Radiotherapy in the treatment of primary central nervous system lymphoma (PCNSL). Journal of Neuro-Oncology. 1999;43(3):241-7.

2. Reni M, Ferreri AJ, Garancini MP, Villa E. Therapeutic management of primary central nervous system lymphoma in immunocompetent patients: results of a critical review of the literature. Annals of Oncology. 1997;8(3):227-34.

3. Abrey LE, DeAngelis LM, Yahalom J. Long-term survival in primary CNS lymphoma. Journal of Clinical Oncology. 1998;16(3):859-63.

4. Abrey LE, Yahalom J, DeAngelis LM. Treatment for primary CNS lymphoma: the next step. Journal of Clinical Oncology. 2000;18(17):3144-50.

5. DeAngelis LM, Seiferheld W, Schold SC, Fisher B, Schultz CJ. Combination chemotherapy and radiotherapy for primary central nervous system lymphoma: Radiation Therapy Oncology Group Study 93-10. J Clin Oncol. 2002;20(24):4643-8.

6. Ferreri AJ, Abrey LE, Blay JY, Borisch B, Hochman J, Neuwelt EA, et al. Summary statement on primary central nervous system lymphomas from the Eighth International Conference on Malignant Lymphoma, Lugano, Switzerland, June 12 to 15, 2002. Journal of Clinical Oncology. 2003;21(12):2407-14.

7. Ferreri AJ, Reni M, Villa E. Therapeutic management of primary central nervous system lymphoma: lessons from prospective trials. Annals of Oncology. 2000;11(8):927-37.

8. Bataille B, Delwail V, Menet E, Vandermarcq P, Ingrand P, Wager M, et al. Primary intracerebral malignant lymphoma: report of 248 cases. J Neurosurg. 2000;92(2):261-6.

9. Ferreri AJ, Reni M, Foppoli M, Martelli M, Pangalis GA, Frezzato M, et al. High-dose cytarabine plus high-dose methotrexate versus high-dose methotrexate alone in patients with primary CNS lymphoma: a randomised phase 2 trial. Lancet. 2009.

10. Pfreundschuh M, Kuhnt E, Trumper L, Osterborg A, Trneny M, Shepherd L, et al. CHOP-like chemotherapy with or without rituximab in young patients with good-prognosis diffuse large-B-cell lymphoma: 6-year results of an open-label randomised study of the MabThera International Trial (MInT) Group. Lancet Oncol. 2011;12(11):1013-22.

11. Illerhaus G, Marks R, Ihorst G, Guttenberger R, Ostertag C, Derigs G, et al. High-dose chemotherapy with autologous stem-cell transplantation and hyperfractionated radiotherapy as first-line treatment of primary CNS lymphoma. Journal of clinical oncology : official journal of the American Society of Clinical Oncology. 2006;24(24):3865-70.

12. Illerhaus G, Muller F, Feuerhake F, Schafer AO, Ostertag C, Finke J. High-dose chemotherapy and autologous stem-cell transplantation without consolidating radiotherapy as first-line treatment for primary lymphoma of the central nervous system. Haematologica. 2008;93(1):147-8.

13. Kasenda B, Schorb E, Fritsch K, Finke J, Illerhaus G. Prognosis after high-dose chemotherapy followed by autologous stem-cell transplantation as first-line treatment in primary CNS lymphoma--a long-term follow-up study. Ann Oncol. 2012.

14. Soussain C, Hoang-Xuan K, Taillandier L, Fourme E, Choquet S, Witz F, et al. Intensive chemotherapy followed by hematopoietic stem-cell rescue for refractory and recurrent primary CNS and intraocular lymphoma: Societe Francaise de Greffe de Moelle Osseuse-Therapie Cellulaire. J Clin Oncol. 2008;26(15):2512-8.

15. Soussain C, Suzan F, Hoang-Xuan K, Cassoux N, Levy V, Azar N, et al. Results of intensive chemotherapy followed by hematopoietic stem-cell rescue in 22 patients with refractory or recurrent primary CNS lymphoma or intraocular lymphoma. Journal of Clinical Oncology. 2001;19(3):742-9.

16. Ferreri AJ, Dell'Oro S, Foppoli M, Bernardi M, Brandes AA, Tosoni A, et al. MATILDE regimen followed by radiotherapy is an active strategy against primary CNS lymphomas. Neurology. 2006;66(9):1435-8.

17. Ponzoni M, Issa S, Batchelor TT, Rubenstein JL. Beyond high-dose methotrexate and brain radiotherapy: novel targets and agents for primary CNS lymphoma. Ann Oncol. 2013.

18. Rubenstein JL, Gupta NK, Mannis GN, Lamarre AK, Treseler P. How I treat CNS lymphomas. Blood. 2013;122(14):2318-30.

19. Illerhaus G, Fritsch K, Egerer G, Lamprecht M, von Bubnoff N, Wolf HH, et al. Sequential High Dose Immuno-Chemotherapy Followed by Autologous Peripheral Blood Stem Cell Transplantation for Patients with Untreated Primary Central Nervous System Lymphoma - a Multicentre Study by the Collaborative PCNSL Study Group Freiburg. Blood. 2012;120(21).

20. Rubenstein JL, Hsi ED, Johnson JL, Jung SH, Nakashima MO, Grant B, et al. Intensive Chemotherapy and Immunotherapy in Patients With Newly Diagnosed Primary CNS Lymphoma: CALGB 50202 (Alliance 50202). J Clin Oncol. 2013.

21. Motomura K, Natsume A, Fujii M, Ito M, Momota H, Wakabayashi T. Long-term survival in patients with newly diagnosed primary central nervous system lymphoma treated with dexamethasone, etoposide, ifosfamide and carboplatin chemotherapy and whole-brain radiation therapy. Leuk Lymphoma. 2011;52(11):2069-75.

22. Takasu S, Wakabayashi T, Kajita Y, Hatano N, Hatano H, Usui T, et al. [Effectiveness of DeVIC chemotherapy for recurrent primary central nervous system lymphoma]. No Shinkei Geka - Neurological Surgery. 2000;28(9):789-94.

23. Correa DD, Maron L, Harder H, Klein M, Armstrong CL, Calabrese P, et al. Cognitive functions in primary central nervous system lymphoma: literature review and assessment guidelines. Ann Oncol. 2007;18(7):1145-51.

24. Ferreri AJ, Blay JY, Reni M, Pasini F, Spina M, Ambrosetti A, et al. Prognostic scoring system for primary CNS lymphomas: the International Extranodal Lymphoma Study Group experience. Journal of Clinical Oncology. 2003;21(2):266-72

Flow Chart

| **Visit schedule and assessments^1^** | **Screening**  **day -14 to day 0** | **Regis- tration^2^**  **d 0** | **Induction treatment:**  **Two 3-week treatment cycles^1^** | | **RA I** | **Consolidation treatment^1^**  **HDT-ASCT** | **RA II** | **Follow up** |
| --- | --- | --- | --- | --- | --- | --- | --- | --- |
|  |  |  | **Visit 1** | **Visit 2** |  | **Visit 3** | **EOT** | **year 1**  **after EOT**  **every**  **3 mo***** |
|  |  |  | **day 0 to day 4**  **of cycle 1**** | **day 0 to day 4**  **of cycle 2**** |  | **Start of HDT** | **day+30**  **after**  **EOT/ASCT** |  |
| Informed consent/ Demographic data | X |  |  |  |  |  |  |  |
| Inclusion/exclusion criteria | X |  |  |  |  |  |  |  |
| Registration |  | X |  |  |  |  |  |  |
| Medical history, height | X |  |  |  |  |  |  |  |
| Treatment administration |  |  | X | X |  | X |  |  |
| ECOG Performance Status | X |  | X | X |  | X | X | X |
| Charlson Comorbidity Index | X |  |  |  |  |  |  |  |
| ADL, MMSE, QoL (EORTC QLQ-C30, - BN20)**^3^** | X |  |  |  | X |  | X | X^3^ |
| Neuropsychological battery**^4^** | X |  |  |  |  |  | X | X^3^ |
| Weight | X |  | X | X |  | X |  |  |
| Vital signs, physical + neurological  examination | X* |  | X* | X* |  | X* | X* | X* |
| Hematology**^5^** / clinical chemistry**^6^** | X* |  | X* | X* |  | X* | X* | X* |
| Creatinine, estimated GFR (MDRD) |  |  | X | X |  | X |  |  |
| LDH | X |  |  |  |  |  |  |  |
| Hepatitis B/C serology, HIV-Test | X* |  |  |  |  |  |  |  |
| Whole body plethysmography | X* |  |  |  |  | X* | X* |  |
| Electrocardiography | X* |  |  |  |  | X* | X* |  |
| Echocardiography | X* |  |  |  |  |  |  |  |
| Testicular ultrasound | X* |  |  |  |  |  |  |  |
| Abdominal ultrasound |  |  | X* | X* |  |  |  |  |
| Whole body CT scan**^7^** | X* |  |  |  |  |  |  |  |
| Whole brain MRI assessment by local and central radiology | X |  |  |  | X |  | X | X**^8^** |
| Shipment to central pathology | X |  |  |  |  |  |  |  |
| BM examination | X* |  |  |  |  |  |  |  |
| Slit lamp examination | X |  |  |  | X**^9^** |  | X**^9^** |  |
| CSF examination**^9^** | X |  |  |  | X**^9^** |  | X**^9^** |  |
| Concomitant medication |  | X (see section 7.6) | | | | | | |
| Adverse events (CTCAE) |  | X (see section 11.1 and 11.1.2) | | | | | | |

RA= response assessment; d= day; mo= months; yr= year; EOT= End of study treatment; ADL= Barthel Activities of daily living; LDH= lactate dehydrogenase; BM= bone marrow; for additional details see corresponding numbering;

* Not to be documented in the CRF

** Interval of treatment administration

*** Thereafter annual conduct of ADL, MMSE, QoL (EORTC QLQ-C30, -BN20) and neuropsychological battery is strongly recommended

1. Examinations and sample collection must be performed before treatment administration; delay up to five days. Interval between treatment cycles should be constant;

2. Informed consent must be obtained prior to any study specific (screening) examination;

3. ADL, MMSE, QOL (EORTC QLQ-C30, -BN20) and neuropsychological battery one year after

EOT and thereafter annually is strongly recommended;

4. Hematology: white blood count (WBC), neutrophils, hemoglobin, and platelets;

5. Blood chemistry: creatinine, total bilirubin, ALT, AST, LDH. Gamma-GT only at screening;

6. If CT is suspicious at diagnosis: FDG-PET;

7. Only performed after excluding increased intracranial pressure by brain MRI; cytology and protein examination;

8. In the course of the follow up period central diagnostic radiology assessment has to be performed in addition to local diagnostic radiology assessment only if measurable lesions are present;

9. Only performed if positive at diagnosis, examination until results are negative.
